# Functional Traits Shape Seed–Rodent Interactions in a Subtropical Forest: Insights From Individual‐Based Tracking With Double‐Duplex PIT Tagging

**DOI:** 10.1002/ece3.72409

**Published:** 2025-10-30

**Authors:** Haifeng Gu, Xifu Yang, Rodolfo Dirzo, Zhibin Zhang

**Affiliations:** ^1^ State Key Laboratory of Integrated Management of Pest Insects and Rodents in Agriculture, Institute of Zoology Chinese Academy of Sciences Beijing China; ^2^ Key Laboratory of Southwest China Wildlife Resources Conservation (Ministry of Education), College of Life Science China West Normal University Nanchong China; ^3^ Department of Biology and Earth Systems Science Stanford University Stanford California USA; ^4^ CAS Centre for Excellence in Biotic Interactions University of Chinese Academy of Sciences Beijing China; ^5^ School of Ecology Hainan University Haikou China

**Keywords:** body size, functional traits, pilferage, PIT tagging, seed fates, seed size, seed–rodent interaction

## Abstract

Functional traits of plants and animals play a pivotal role in shaping mutualistic or predatory interactions within plant–animal systems, directly regulating the structure and function of forest ecosystems. Yet, the outcomes of multispecies interactions—particularly in seed–rodent systems—remain inadequately resolved, largely because traditional methods fail to track individual‐level interactions and seed fates with sufficient precision. To address this gap, we applied a novel double‐duplex passive integrated transponder (PIT) tagging technique to investigate the fates of seeds from four sympatric tree species (with distinct seed traits) when exploited by two sympatric rodent species (with contrasting body sizes) in a subtropical forest of Southwest China from 2018 to 2019. Our results revealed that rodent body size and seed size are key determinants of seed fates. The larger rat *Niviventer confucianus* scatter‐hoarded and consumed seeds of all four trees, with a significant preference for large‐sized seeds of *Quercus variabilis* and *Lithocarpus harlandii*. In contrast, the smaller mouse *Apodemus draco* did not hoard the large‐sized seeds of *L. harlandii* and showed a significant preference for small‐sized seeds of *Camellia oleifera*. Additionally, *N. confucianus* exhibited a higher interspecific pilfering rate on seeds of *C. oleifera* and *L. harlandii* than *A. draco* . Our study highlights the significant role of size traits in shaping the mutualistic or predatory interactions in seed–rodent systems and demonstrates the utility of individual‐based tracking in disentangling complex species interactions.

## Graphical abstract

Using double‐duplex PIT tagging, this study examines how seed and rodent size traits shape interactions between four tree species and two‐rodent species in a subtropical forest, finding larger rodents prefer large seeds, whereas smaller ones favor small seeds, with size‐related behaviors structuring mutualism‐predation balances. These results highlight the role of functional trait diversity in ecological interactions and demonstrate the utility of individual‐level tracking for disentangling complex species relationships.

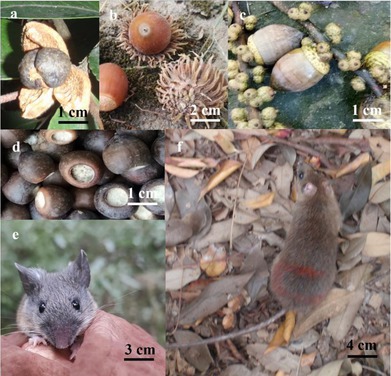

## 1 Introduction

Mutualistic and predatory interactions between animals and plants are central to ecosystem structure and function (Vazquez et al. 2005; Bastolla et al. 2009; Jordano 2010; Li et al. 2024). In ecological communities, these interactions are often diffuse, involving combined effects of multiple species (Forget and Vander Wall 2001; Bronstein et al. 2006; Bascompte and Jordano 2007; Vander Wall and Beck 2012; Yamawo and Ohno 2024). Seed–rodent interactions are typical examples of such diffuse interactions, as multiple coexisting rodent species exploit seeds from multiple coexisting plant species (Vander Wall and Beck 2012; Zwolak et al. 2024; Yang, Zhao, Ma, et al. 2025).

In forest ecosystems, the interactions between rodents and seeds have both antagonistic and mutualistic aspects, driven by the contrasting effects of seed scatter‐hoarding and predation of rodents (Jansen et al. 2012; Zwolak and Crone 2012; Lichti et al. 2014; Zhang et al. 2015; Bogdziewicz et al. 2020). Scatter‐hoarding rodents establish many small caches, each containing one or a few seeds, near the soil surface, and this behavior can promote seedling recruitment (Vander Wall 1990; Niu et al. 2020; Yang et al. 2023). On the contrary, these rodents also act as predators by consuming or storing them in larder hoards, which is harmful to seedling establishment (Vander Wall et al. 2001; Zhang et al. 2021). The balance between mutualism and predation in seed–rodent interactions is determined by the extent of scatter‐hoarding relative to seed consumption (Gomez et al. 2008; Zeng et al. 2021; Zwolak et al. 2022), and this balance can range from mutualism to antagonism across different pairs of seed and rodent species (Schupp et al. 2010; Wandrag et al. 2013; Zhang et al. 2021; van Leeuwen et al. 2022). These interaction patterns are often influenced by the functional traits (such as morphological, life‐history, and physiological traits) of both rodents and seeds (Chang and Zhang 2014; Zhang et al. 2016; Steele 2021; Lai et al. 2024).

Functional traits of both animals and plants are crucial in shaping the structure of mutualistic or predatory interaction networks (Jordano et al. 2003; Dirzo et al. 2007; Zhang et al. 2015; Wang, Tai, et al. 2024; Wu et al. 2024). Trait matching governs species interactions by regulating niche breadth and partitioning in ecosystems (Hillebrand and Matthiessen 2009; Vazquez et al. 2009; Albrecht et al. 2018). Similar to other plant–animal interactions, the structure of seed–rodent interactions involving multiple partners is associated with functional traits, such as seed size, nutrition, seed coat thickness, tannin levels, and rodent body size (Chang and Zhang 2014; Zhang et al. 2015; Yang, Zhao, Ma, et al. 2025). Among these traits, seed size and rodent body size have been identified as ecologically and evolutionarily important across diverse ecosystems (Dirzo et al. 2007; Galetti et al. 2013; Chang and Zhang 2014; Zhang et al. 2015; Yu et al. 2025). Yet, critical questions remain unanswered: how do these functional traits determine whether rodents act primarily as seed dispersers (beneficial to plant recruitment) or predators (detrimental to seed survival)? And how do these traits modulate the continuum between mutualism and antagonism in seed–rodent interactions within natural ecosystems (Zhang et al. 2021, 2024)? These knowledge gaps hinder our ability to predict how shifts in rodent or plant communities might alter forest regeneration dynamics.

Traditional methods for studying seed–rodent interactions have hindered investigations into the mechanisms underlying the effects of functional traits. Various seed‐marking methods exist, including radioactive isotope marking (Vander Wall et al. 2006), magnet or metal marking (Stapanian and Smith 1978; Sork 1984), thread marking (Schupp 1988; Forget 1990), color marking (Lemke et al. 2009), active radio marking (Tamura 1994; Jansen et al. 2012), and label tagging (Xiao et al. 2006; Yang et al. 2020; Yang, Zhao, and Zhang 2025). For rodents, marking methods include freeze branding (Hadow 1972), active radio marking (Jansen et al. 2012), and color marking (Gu et al. 2017). However, few studies have used these methods to identify seed–rodent interactions at the species or individual level, limiting our understanding of the natural history of these relationships and the extent of mutualism or antagonism (Jansen et al. 2012; Gu et al. 2017). Even fewer studies have overcome methodological limitations to enable automatic identification of seeds, rodents, and details of their interactions.

To address these gaps, we used the double‐duplex PIT tagging method (as described in Gu et al. 2021), which allows for high‐accuracy and high‐efficiency tracking of individual‐level seed–rodent interactions. This technique enables simultaneous identification of individual rodents (via half‐duplex PIT tags) and individual seeds (via full‐duplex PIT tags) as they interact, providing detailed data on seed harvesting, dispersal, hoarding, consumption, and pilferage.

In this study, we examined differences in seed fates among four sympatric tree species (*Camellia oleifera*, *Quercus variabilis* , *Lithocarpus harlandii*, and *L. hancei*) with contrasting seed traits, under predation of two dominant rodent species ( *Niviventer confucianus* and *Apodemus draco* ) with contrasting body sizes, in a subtropical forest in Southwest China from 2018 to 2019. We tested the hypothesis that larger rodent species would prefer to consume and scatter‐hoard large‐sized seeds with hard coats, whereas smaller rodent species would prefer to consume and scatter‐hoard small‐sized seeds with soft coats. By using individual‐based tracking, we aimed to disentangle how size‐related traits shape seed–rodent interactions and their outcomes.

## 2 Materials and Methods

### 2.1 Study Site and Species

Our study was carried out in a secondary subtropical forest located near Dujiangyan City, Sichuan Province, Southwest China (31°03′ N, 103°43′ E, elevation ranging from 700 to 1000 m). The region has an average annual temperature of 10°C and annual precipitation between 1300 and 1800 mm. Local common tree species include *Castanopsis fargesii*, *Camellia oleifera*, *Quercus variabilis* , *Lithocarpus harlandii*, *Acer catalpifolium*, *Q. serrata* , *L. hancei*, *Phoebe zhennan*, and *Q. glauca* (Yang et al. 2018). Seeds of these plants are consumed or dispersed by local rodent species, including *Apodemus draco* , *Niviventer confucianus* , *A. chevrieri* , *N. fulvescens* , and *Leopoldamys edwardsi* (Yang et al. 2020; Yang, Han, et al. 2022). Our 0.5‐ha study plot was selected on a hilltop covered by a typical secondary forest patch in the area.

**FIGURE 1:**
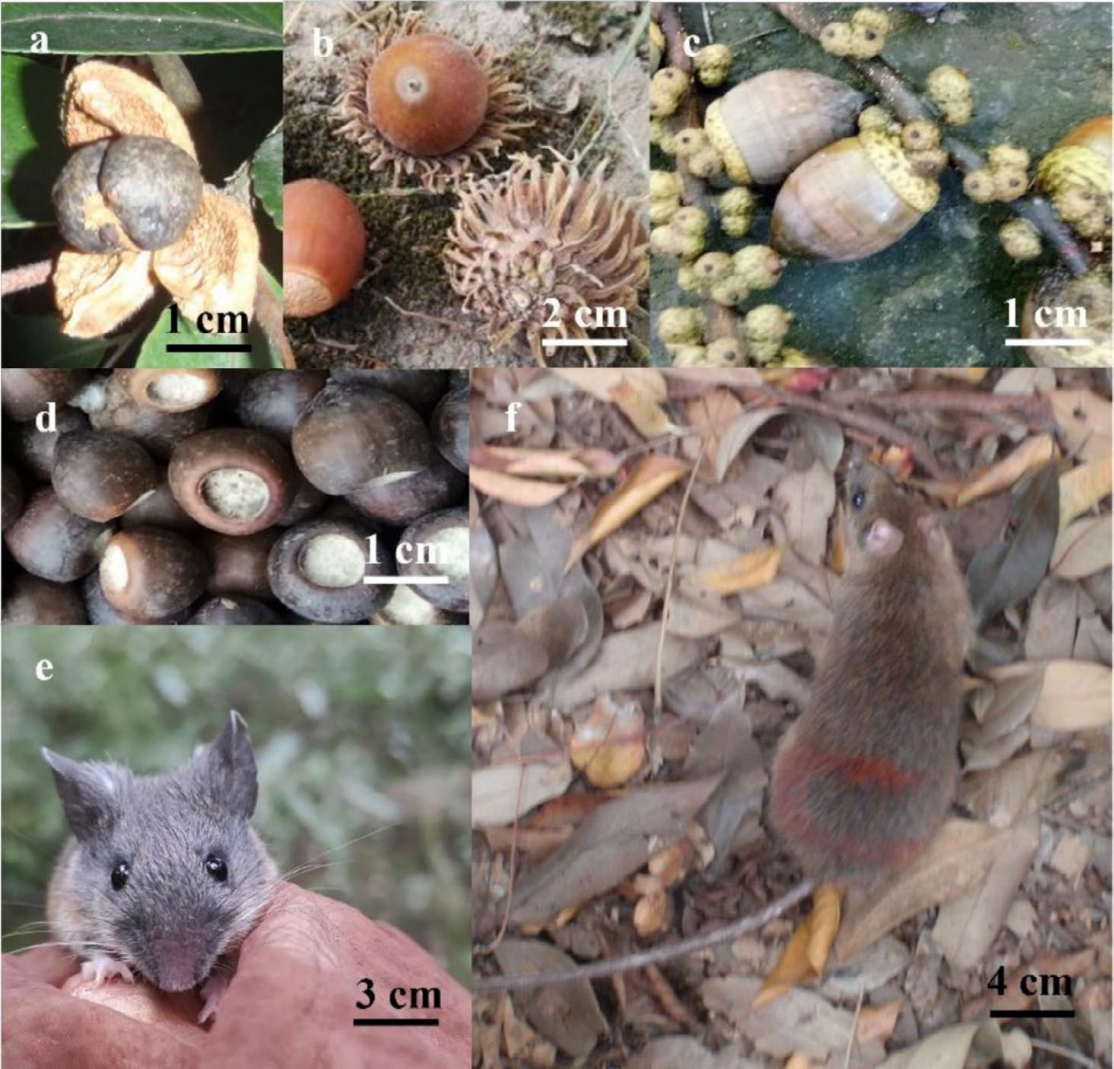
Seed and rodent species involved in this study are as follows. Seed species: (a) Seeds of *Camellia oleifera* (photo credits: Haifeng Gu); (b) Acorns of *Quercus variabilis* (photo credits: Haifeng Gu); (c) Acorns of *Lithocarpus harlandii* (photo credits: Haifeng Gu); and (d) Acorns of *L. hancei* (photo credits: Haifeng Gu). Rodent species: (e) An *Apodemus draco* individual (photo credits: Xifu Yang); and (f) A *Niviventer confucianus* individual (photo credits: Haifeng Gu). These species were selected due to their abundance in the subtropical forest study area and their documented roles in seed–rodent interactions, with distinct traits (e.g., seed size, coat thickness, rodent body mass) that facilitate investigating trait‐mediated ecological relationships.

We selected seeds from four tree species—oil tea ( *C. oleifera* ), Chinese cork oak ( *Q. variabilis* ), Harlandy's stone oak (*L. harlandii*), and Hance's stone oak (*L. hancei*)—to investigate differences in scatter‐hoarding behavior between two sympatric rodent species: *N. confucianus* (Chinese white‐bellied rat) and *A. draco* (South China field mouse), which differ in body mass and food preference (Figure 1; Gu et al. 2021). These tree species are abundant in the subtropical forests of Southwest China. *C. oleifera* produces capsules containing 1–10 seeds; *Q. variabilis* produces globose acorns; *L. harlandii* produces the biggest and toughest acorns in the study region; *L. hancei* produces small globose acorns with husks much tougher than those of *Q. variabilis* and *C. oleifera* . The four‐seed species differ significantly in key functional traits, detailed in Table 1. All four tree species are food sources for local rodents (Yang et al. 2018; Yang, Gu, et al. 2022).

**TABLE 1:** Differences in seed traits among four‐seed species: *Camellia oleifera* (Co), *Quercus variabilis* (Qv), *Lithocarpus harlandii* (Lhar), and *L. hancei* (Lhan).

Species	*n*	Weight (mean ± SD, g)	Seed coat thickness (mm)	Starch (%)	Fat (%)	Tannin (%)
Co	184	1.37 ± 0.39	0.33 ± 0.09	1.87	52.92	0.10
Qv	184	3.49 ± 0.93	0.56 ± 0.09	49.50	2.54	6.69
Lhar	184	5.30 ± 1.03	1.43 ± 0.20	53.7	0.58	0.97
Lhan	184	2.24 ± 0.42	0.76 ± 0.11	52.59	1.15	1.51

### 2.2 Tagging Methods for Rodents and Seeds

Field experiments were carried out from March 2018 to January 2019 within a 0.5‐ha gridded plot, where we established a trapping grid with 66 live traps (25 × 12 × 10 cm) spaced at 10 m intervals. Each trap was baited with a fresh seed of *Castanea mollissima* (Chinese chestnut)—a locally abundant species selected for its low tannin content (high palatability to rodents) and proven effectiveness: Our prior work (Yang, Zhao, and Zhang 2025) using this bait achieved a 17.5% capture rate in regional surveys, confirming its ability to attract target rodent species. Traps were checked and reset daily between 07:00 and 08:00 to minimize rodent stress from prolonged captivity.

Each captured rodent was identified to species, weighed (to the nearest 0.1 g), fitted with a half‐duplex (HDX) PIT tag (12 mm length × 1 mm diameter, < 0.1 g; Biomark Inc., USA), and dye‐marked. Tag implantation followed the protocol described by Korslund and Steen (2006): tags were subcutaneously inserted under the dorsal neck skin (a location minimizing interference with movement or grooming) using sterile 18‐gauge needles. All tools were sterilized with 75% ethanol to prevent infection, and the procedure took < 30 s per individual to reduce handling stress.

To ensure PIT tagging did not adversely affect rodent health or behavior, we implemented rigorous validation: (1) The tag weight represented < 0.6% of the average body mass of *A. draco* (the smaller species, 18.1 ± 4.6 g, mean ± SD) and < 0.19% of *N. confucianus* (53.1 ± 11.5 g)—well below the 1%–2% body mass threshold widely accepted to avoid disrupting small mammal locomotion, foraging, or energy metabolism (Korslund and Steen 2006; Gu et al. 2021). (2) Over the 11‐month study, no signs of injury (e.g., inflammation, tag expulsion), abnormal behavior (reduced foraging, altered movement), or mortality were observed in tagged individuals. Pre‐trapping observations of untagged rodents confirmed baseline seed‐handling patterns, and post‐tagging monitoring of tagged individuals showed consistency in key behaviors (e.g., harvest rate, dispersal distance, and hoarding frequency)—aligning with prior studies demonstrating that lightweight PIT tags do not interfere with rodent seed‐related behaviors (Gu et al. 2021).

Each tagged individual's PIT serial number was recorded, and unique dye marks were applied using wine‐red human hair dye (Gu et al. 2017) before releasing them at the capture site. Trapping was terminated after three successive days with no new untagged captures. Animal handling in the experiments was supervised by the Animal Ethics and Welfare Committee of the Institute of Zoology, Chinese Academy of Sciences (permission: IOZ13034) and was in accordance with the international guides of wild animal ethics. We tagged 32 individuals of the two dominant species: 13 *A. draco* and 19 *N. confucianus* , with *N. confucianus* showing higher trap success (Table S1). Two *Niviventer fulvescens* (Chestnut white‐bellied rat) were occasionally captured but excluded due to a small sample size (*n* = 2), insufficient for analyzing seed‐handling behaviors. Focusing on *A. draco* and *N. confucianus* (32 individuals) ensured robust inference about size‐related seed–rodent interactions.

We collected 736 mature seeds from four species*—C. oleifera*, *Q. variabilis* , *L. harlandii*, and *L. hancei* (184 seeds per species)—from nearby forest patches. Each seed was weighed and tagged with a 10‐cm‐long thin steel wire and a pink plastic label (2.5 × 3.6 cm, < 0.1 g) inscribed with seed ID and placement date, following the standard seed tagging protocols (Xiao et al. 2006; Yu et al. 2023). All plastic labels were cut into 16 distinct shapes (Gu et al. 2021). A full‐duplex (FDX) PIT tag (12 mm in length, 1 mm in diameter, < 0.1 g; Biomark Inc., USA) was attached to each tag label with Sellotape, and the tag's serial number was recorded. This allowed all seeds to be identified via both infrared (IR) cameras and PIT readers.

### 2.3 Seed Placement and Monitoring at Seed Stations

A 1‐m^2^ seed station was established at the center of the study plot, with leaf litter cleared to expose the soil surface. Once rodent trapping was completed, batches of 16 double‐tagged seeds (four seeds from each of the four species: *C. oleifera* , *Q. variabilis* , *L. harlandii*, and *L. hancei*) were placed evenly in a circular pattern on the station's ground. New seeds were added or replaced when the existing batch was either removed, consumed in situ by rodents, or left untouched for five consecutive days.

**FIGURE 2:**
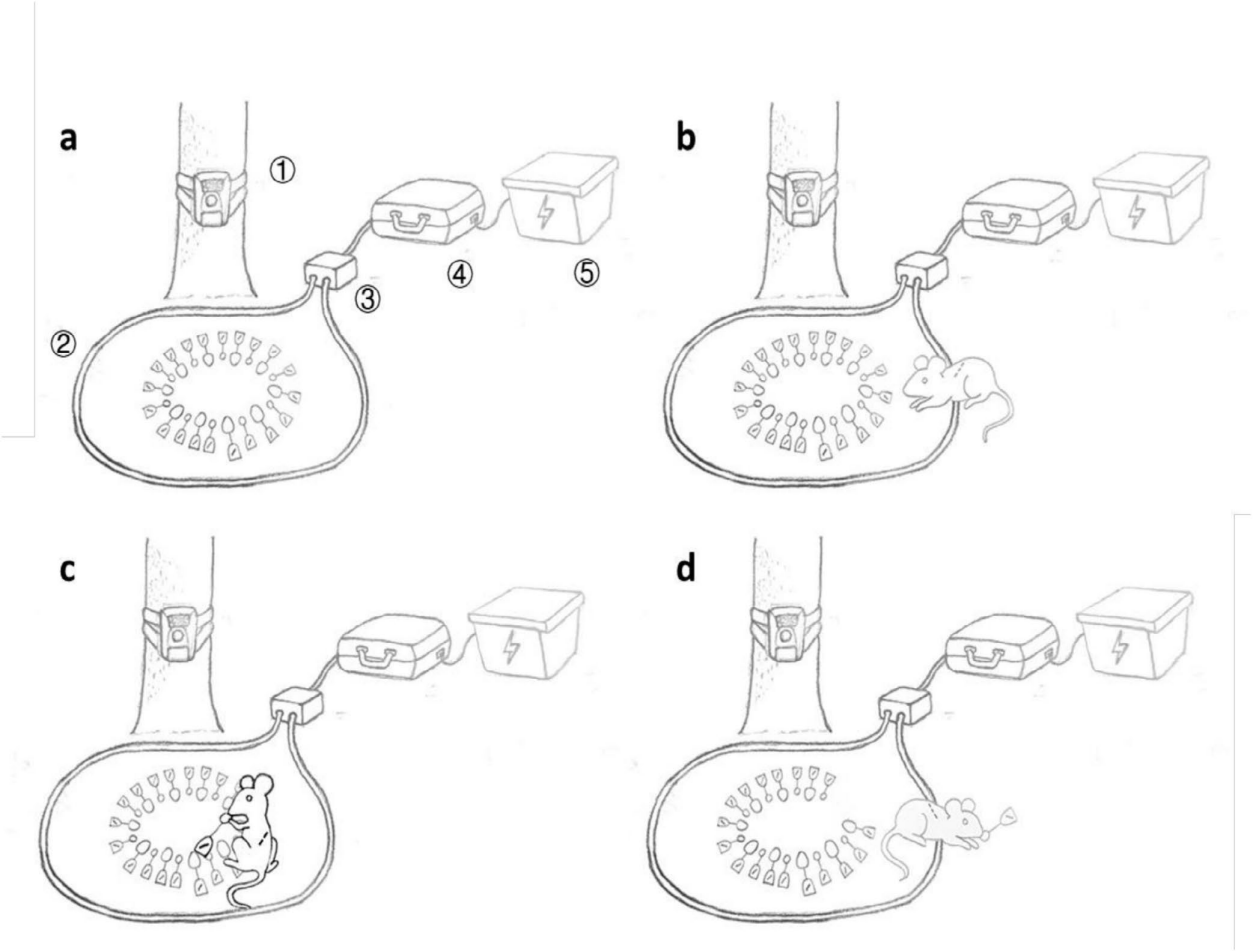
Illustration of the operating principles of the double‐duplex passive integrated transponder (PIT) tagging and monitoring system. (a) a seed station with 20 full‐duplex PIT tagged seeds placed near a tree. ① an IR camera; ② the antenna circling the seeds; ③ the junction box; ④ the console of the modified PIT reader, capable of reading full‐duplex and half‐duplex PIT tags simultaneously; ⑤ the power station, home‐made with a plastic storage box and two 12 V automobile storage batteries in series; (b) a half‐duplex PIT tagged rodent approaching the seed station and crossing over the antenna on the ground while the reader records its identity; (c) the tagged rodent eats a tagged seed in situ and is recorded by the IR camera; (d) the tagged rodent disperses a tagged seed and crosses the antenna again to leave the seed station. The reader records the identities of both the rodent and the dispersed seed. For details, refer to Gu et al. (2021).

At the seed station, a PIT reader console and loop antenna were connected via a junction box (Biomark Inc., USA), with a section of the antenna formed into a circle (approximately 1 m in radius) that encircled the station (Figure 2). When an HDX‐tagged rodent crossed this antenna to harvest tagged seeds at the station, its identity (via PIT tag serial number) was automatically recorded by the reader. When a rodent carrying an FDX‐tagged seed in its mouth departed the station, it crossed the antenna again, triggering the automatic recording of both the rodent's and the seed's identities. Additionally, an IR camera (Little Acorn Outdoors, Green Bay, USA) was positioned at the seed station to document instances where rodents consumed seeds within the station area without crossing the antenna. In such cases, interactions between the consumed seeds and their predators could be identified using the shapes of the plastic seed labels captured in video footage, combined with rodent identities recorded by the PIT reader (Figure 2).

### 2.4 Tracking of Dispersed Tagged Seeds and Definitions of Seed Fates

Each day between 0800 and 1600 h, we searched the area within a 30 m radius of the seed station to locate dispersed seeds (for details on the selection of this searching radius, see Gu et al. 2017). The pink seed labels attached to seeds facilitated easy retrieval of dispersed seeds (Xiao et al. 2006). For each retrieved seed, we recorded the dispersal date, its fate (as defined below), and specific details of the cache site, including burial depth, substrate type, and microhabitat characteristics.

An infrared camera was placed near each scatter‐hoarded seed to monitor its subsequent fates—whether it was re‐cached by the original cache owner or pilfered by conspecific or interspecific rodents. Individual rodents involved in dispersing or consuming cached seeds were identified using their unique dorsal dye marks in the camera footage (for rodent tagging methods, refer to Section 2.2). Camera monitoring ceased once all cached seeds had either been consumed or classified as missing.

To standardize the classification of seed fates, we defined the following categories based on the entire process of seed handling by rodents (Gu et al. 2021):

1. Initially harvested seeds (IHS): seeds that were either consumed or removed by rodents from the seed station, encompassing seeds eaten in situ (EIS), seeds eaten after removal (EAR), seeds scatter‐hoarded during primary dispersal (SH_I_), and missing seeds (M). Calculated as: IHS = EIS + EAR + SH_I_ + M.
2. Initially scatter‐hoarded seeds (SH_I_): Seeds that were scatter‐hoarded by rodents under the soil surface or with leaf litter during primary dispersal (i.e., the first movement away from the seed station). This category includes seeds retained by the original cache owners (RSH; either consumed by owners or not pilfered by the end of the experiment), seeds pilfered by conspecifics (CL), and seeds pilfered by interspecific rodents (IL). Calculated as: SH_I_ = RSH + CL + IL.
3. Retained scatter‐hoarded seeds (RSH): Initially scatter‐hoarded seeds that remained under the control of the original cache owners (either consumed or protected) until the end of the experiment, reflecting the benefits of scatter‐hoarding to the cache owner.
4. Finally scatter‐hoarded seeds (SH_F_): Seeds that remained scatter‐hoarded by the end of the experiment, including RSH, seeds pilfered from conspecific caches (CP), and seeds pilfered from interspecific caches (IP). Calculated as: SH_F_ = RSH + CP + IP.
5. Finally harvested seeds (FHS): The total number of seeds confirmed to have been either retained or consumed by rodents by the end of the experiment. Calculated as: FHS = SH_F_ + EAR + EIS.

### 2.5 Data Analysis

All data analyses were conducted with R 4.4.0 (R Core Team 2024). To test for significant differences in proportions—including interspecific differences between the two‐rodent species in terms of foraging preference (seed harvesting, scatter‐hoarding, and consumption) and derived benefits (from scatter‐hoarding)—we used 2 × 2 contingency tables and Pearson's chi‐squared tests (Gu et al. 2021). Fisher's exact tests were applied when the expected value of any cell in the contingency table was smaller than 5.

## 3 Results

### 3.1 Overall Seed Fate

**FIGURE 3:**
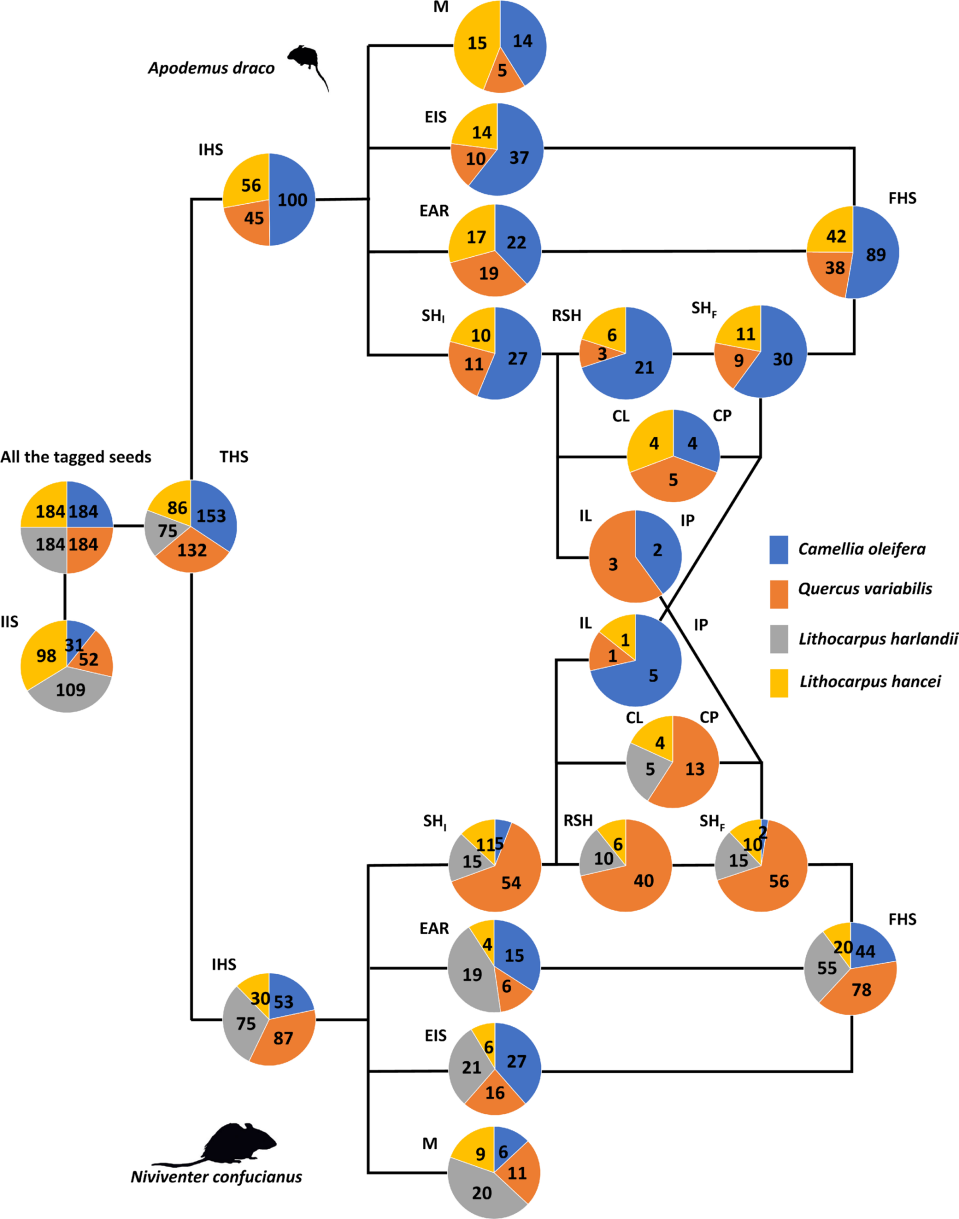
Seed fates of *Camellia oleifera*, *Quercus variabilis* , *Lithocarpus harlandii*, and *L. hancei* harvested by *Apodemus draco* and *Niviventer confucianus* during the experiment. The numbers within each pie chart indicate the count of seeds corresponding to each fate category. CL, conspecific losses of scatter‐hoarded seeds; CP, conspecific pilfered seeds; EAR, seeds eaten after removal; EIS, seeds eaten in situ; FHS, finally harvested seeds; IHS, initially harvested seeds; IIS, seeds remain intact in situ; IL, interspecific losses of scatter‐hoarded seeds; IP, interspecific pilfered seeds; M, missing seeds; RSH, scatter‐hoarded seeds retained by cache owners; SH_F_, finally scatter‐hoarded seeds; SH_I_, initially scatter‐hoarded seeds; THS, total harvested seeds. For additional details on these abbreviations, refer to Section 2.4.

Of 736 tagged seeds, 60.6% (446 seeds) were harvested (dispersed or consumed) by rodents; the remaining seeds untouched by rodents were retrieved by researchers (Figure 3). Among the harvested seeds, 233 seeds (52.2%) were eaten, either in situ or after removal from the seed station, and 133 seeds (29.8%) were initially scatter‐hoarded. A small proportion (17.9%) of the harvested seeds was classified as missing, with their final fates undetermined.

From the perspective of seed species, the harvesting rate varied: 83.2% of *C. oleifera* seeds, 71.7% of *Q. variabilis* seeds, 40.8% of *L. harlandii* seeds, and 46.7% of *L. hancei* seeds were harvested by rodents. This variation in harvesting rates aligns with the contrasting preferences of the two‐rodent species, with the highly preferred *C. oleifera* (by *A. draco* ) and *Q. variabilis* (by *N. confucianus* ) showing higher overall harvest rates, whereas the less preferred *L. harlandii* and *L. hancei* had lower harvest rates (Figure 3).

Additionally, of the initially scatter‐hoarded seeds (SH_I_), 64.7% were retained by cache owners (RSH), 26.3% were lost to conspecific pilferers (CL), and 9.0% were lost to interspecific pilferers (IL), indicating that pilferage is a notable factor affecting the fate of cached seeds (Figure 3). The proportion of finally scatter‐hoarded seeds (SH_F_) relative to IHS also varied by seed species: *Q. variabilis* showed the highest proportion (49.2%), followed by *L. harlandii* (25.6%), whereas *C. oleifera* and *L. hancei* had lower proportions (20.9% and 20.0%, respectively). This pattern reflects the differential effectiveness of scatter‐hoarding across seed species, aligning with the size‐related preferences of the two‐rodent species.

### 3.2 Interspecific Differences in Seed Harvesting, Consumption, and Scatter‐Hoarding

**FIGURE 4:**
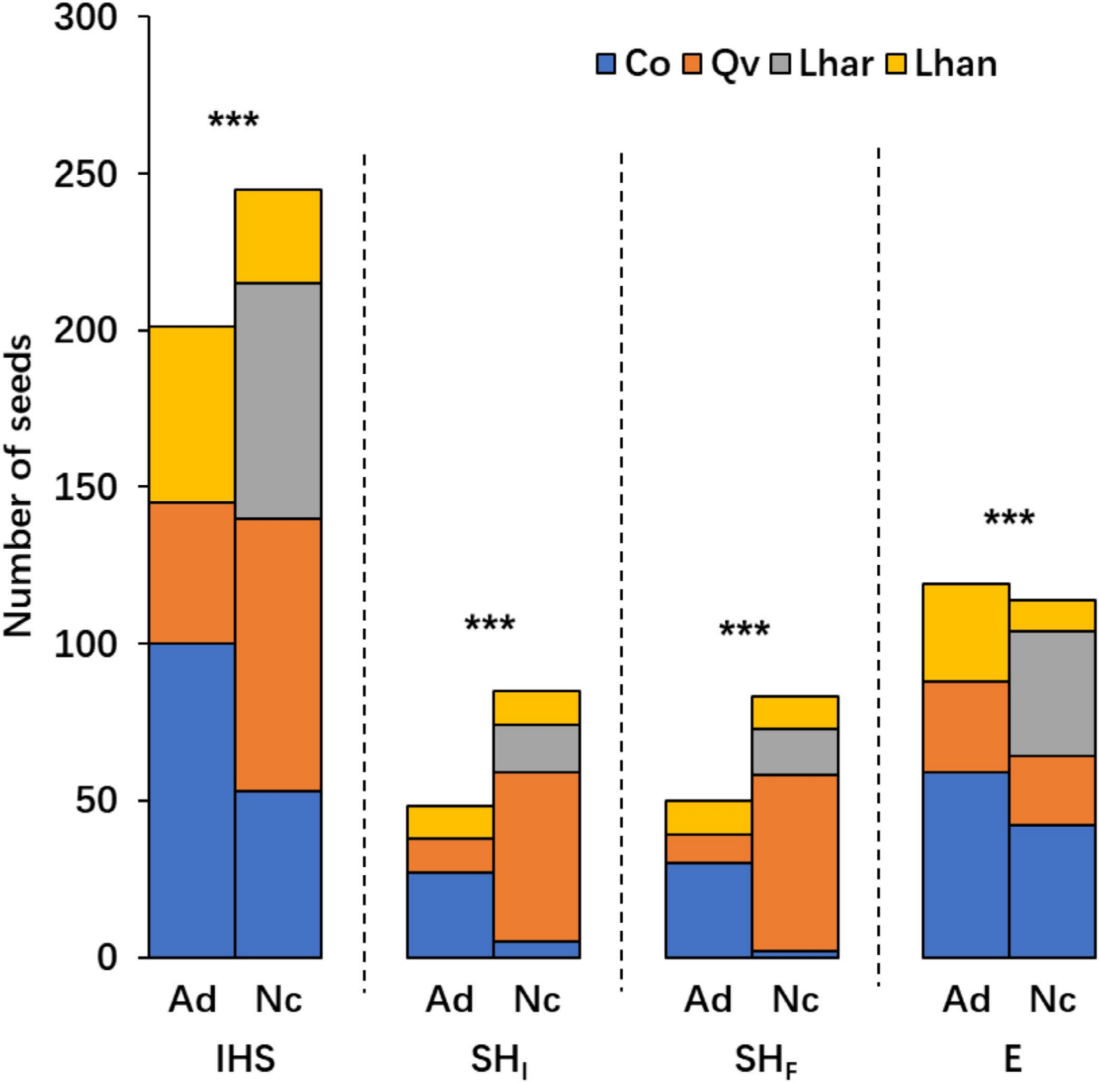
Contrasting patterns of initial harvesting, scatter‐hoarding (both initial and final), and consumption preference between the smaller *Apodemus draco* (Ad) and the larger *Niviventer confucianus* (Nc) across seeds of *Camellia oleifera* (blue), *Quercus variabilis* (orange), *Lithocarpus harlandii* (gray), and *L. hancei* (yellow). E, seeds eaten by the rodents (including those eaten in situ and after removal from the seed station); IHS, initially harvested seeds; SH_F_, finally scatter‐hoarded seeds; SH_I_, initially scatter‐hoarded seeds. For detailed explanations of abbreviations, see Section 2.4. For comprehensive statistical analyses, refer to Table S2. *** indicates *p* < 0.001.

*A. draco* and *N. confucianus* harvested 45.1% and 54.9% of 446 IHS, respectively (Figure 4). The 201 IHS harvested by *A. draco* included 100 *C. oleifera* , 45 *Q. variabilis* , and 56 *L. hancei*, whereas the remaining 226 IHS harvested by *N. confucianus* included 53 *C. oleifera* , 87 *Q. variabilis* , 75 *L. harlandii*, and 30 *L. hancei*. The smaller *A. draco* showed a marked preference (49.8%) for harvesting seeds of *C. oleifera* , which were the smallest among all four seed species (*χ* ^2^ = 100.71, *p* < 0.001), whereas the larger *N. confucianus* displayed a significant preference for harvesting the seeds of *Q. variabilis* (38.5%) and *L. harlandii* (33.2%), which were much bigger than the other two seed species (*χ* ^2^ = 30.97, *p* < 0.001; Table S2). Harvesting preferences differed significantly between species (*χ* ^2^ = 107.37, *p* < 0.001).

*A. draco* and *N. confucianus* scatter‐hoarded 36.1% and 63.9% of 133 initially scatter‐hoarded seeds (SH_I_), respectively (Figure 4). Forty‐eight SH_I_ scatter‐hoarded by *A. draco* included 27 *C. oleifera* , 11 *Q. variabilis* , and 10 *L. hancei*, whereas the rest 85 SH_I_ scatter‐hoarded by *N. confucianus* included 5 *C. oleifera* , 54 *Q. variabilis* , 15 *L. harlandii*, and 11 *L. hancei*. The smaller *A. draco* showed a strong preference (56.3%) for initially scatter‐hoarding seeds of *C. oleifera* (*χ* ^2^ = 31.17, *p* < 0.001), whereas *N. confucianus* exhibited a significant preference (63.5%) for initially scatter‐hoarding the seeds of *Q. variabilis* (*χ* ^2^ = 69.68, *p* < 0.001; Table S2). There was a significant difference between the two species in their initial scatter‐hoarding preferences (*χ* ^2^ = 52.38, *p* < 0.001; for *C. oleifera* , *χ* ^2^ = 39.88, *p* < 0.001; for *Q. variabilis* , *χ* ^2^ = 18.66, *p* < 0.001).

*A. draco* and *N. confucianus* scatter‐hoarded 37.6% and 62.4% of 133 finally scatter‐hoarded seeds (SH_F_), respectively (Figure 4). Fifty SH_F_ scatter‐hoarded by *A. draco* included 30 *C. oleifera* , nine *Q. variabilis* , and 11 *L. hancei*, whereas the rest 83 SH_F_ scatter‐hoarded by *N. confucianus* included two *C. oleifera* , 56 *Q. variabilis* , 15 *L. harlandii*, and 10 *L. hancei*. *A. draco* showed a marked preference (60.0%) for finally scatter‐hoarding seeds of *C. oleifera* (*χ* ^2^ = 38.16, *p* < 0.001), whereas *N. confucianus* showed a strong preference (67.5%) for finally scatter‐hoarding the seeds of *Q. variabilis* (*χ* ^2^ = 83.99, *p* < 0.001; Table S2). The final scatter‐hoarding preference of the two species differed significantly (*χ* ^2^ = 69.63, *p* < 0.001; for *C. oleifera* , *χ* ^2^ = 53.53, *p* < 0.001; for *Q. variabilis* , *χ* ^2^ = 28.61, *p* < 0.001).

Regarding seed consumption, *A. draco* and *N. confucianus* consumed 51.1% and 48.9% of the 233 eaten seeds (E)—including seeds eaten in situ (EIS) and after removal (EAR)—respectively (Figure 4). The 119 E consumed by *A. draco* included 59 *C. oleifera* , 29 *Q. variabilis* , and 31 *L. hancei*, whereas the rest 114 E consumed by *N. confucianus* included 42 *C. oleifera* , 22 *Q. variabilis* , 40 *L. harlandii*, and 10 *L. hancei*. *A. draco* showed a significant preference (49.6%) for consuming seeds of *C. oleifera* (*χ* ^2^ = 58.58, *p* < 0.001), whereas *N. confucianus* preferred consuming seeds of *C. oleifera* (36.8%) and *L. harlandii* (35.1%) (*χ* ^2^ = 24.53, *p* < 0.001; Table S2). The consumption preferences of the two species differed significantly (*χ* ^2^ = 54.50, *p* < 0.001).

### 3.3 Interspecific Differences in Seed Pilferage on Initially Scatter‐Hoarded Seeds

Pilferage was categorized as conspecific (CP) or interspecific (IP). We calculated: conspecific pilferage benefits (CPB = CP/SH_F_), interspecific pilferage benefits (IPB = IP/SH_F_), and total pilferage benefits (TPB = (CP + IP)/SH_F_) (Table S3).

**FIGURE 5:**
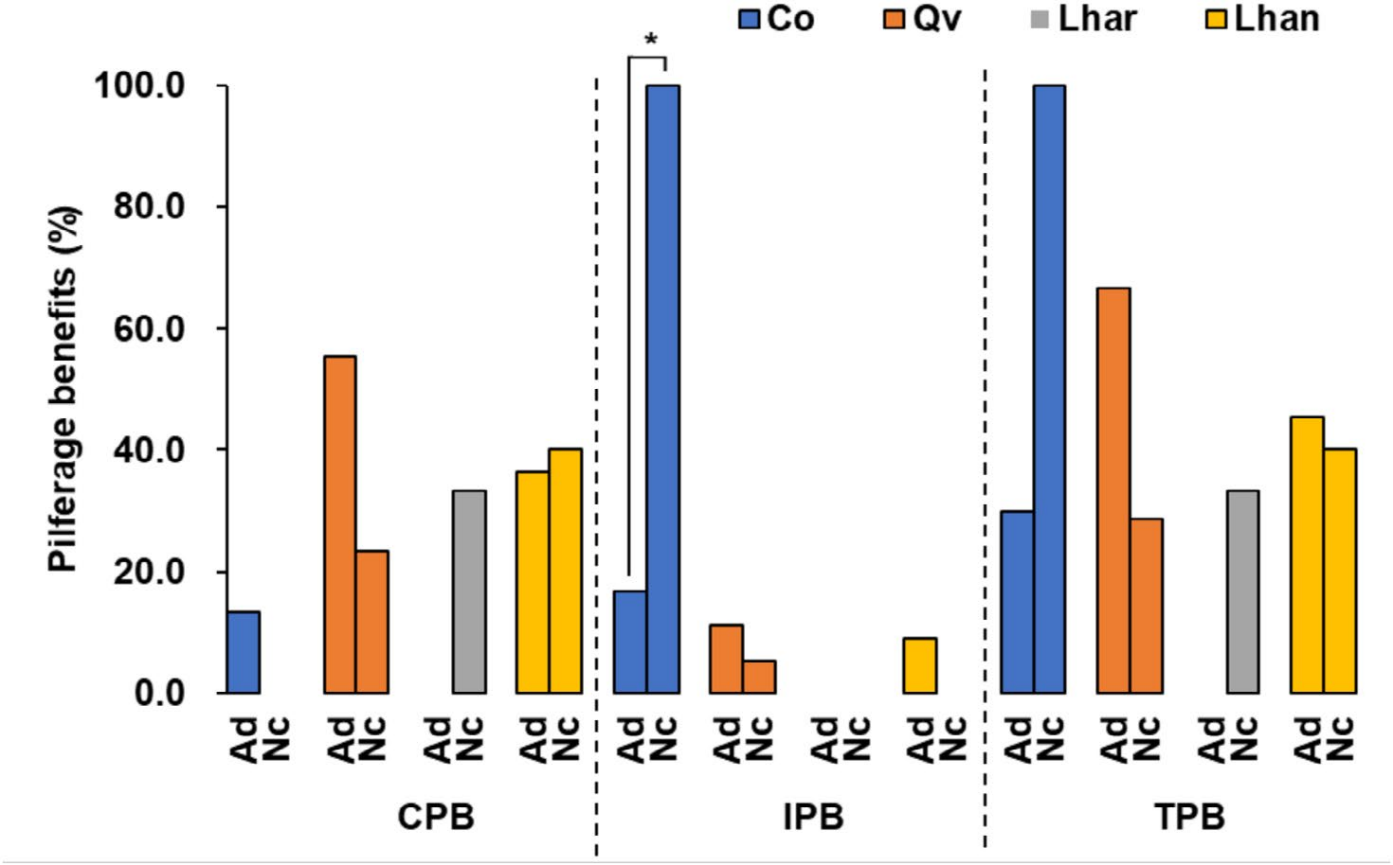
Interspecific difference in the benefits derived from seed pilferage (CPB, IPB, and TPB) between the smaller *Apodemus draco* (Ad) and the larger *Niviventer confucianus* (Nc) across seeds of *Camellia oleifera* (blue), *Quercus variabilis* (orange), *Lithocarpus harlandii* (gray), and *L. hancei* (yellow). CP, conspecific pilfered seeds; CPB, conspecific pilferage benefits (CP/SH_F_); IP, interspecific pilfered seeds; IPB, interspecific pilferage benefits (IP/SH_F_); SH_F_, finally scatter‐hoarded seeds; TP, total pilfered seeds; TPB, total pilferage benefits (TP/SH_F_). For detailed explanations of these abbreviations, see Section 2.4. For comprehensive statistical analyses, refer to Table S4. *, indicates 0.01 < *p* < 0.05.

No significant differences in CPB between rodent species were found for *C. oleifera* (Fisher's exact test: *p* = 1.000), *Q. variabilis* (*p* = 0.101), or *L. hancei* (*p* = 1.000) (Tables S3 and S4). *N. confucianus* had higher CPB for *L. harlandii* (as *A. draco* did not pilfer these seeds; Figure 5).

*N. confucianus* had higher IPB for *C. oleifera* than *A. draco* (Fisher's exact test: *p* = 0.042). No significant differences were found for *Q. variabilis* (*p* = 0.458), *L. harlandii* (no interspecific pilferage), or *L. hancei* (*p* = 1.000) (Figure 5; Table S4).

No significant differences in TPB between species were found for *C. oleifera* (Fisher's exact test: *p* = 0.111), *Q. variabilis* (*χ* ^2^ = 3.47, *p* = 0.063), or *L. hancei* (*p* = 1.000) (Table S4). *N. confucianus* had higher TPB for *L. harlandii* (as *A. draco* did not pilfer these seeds; Figure 5).

## 4 Discussion

Our findings indicate that the fates of seeds from four coexisting tree species are closely linked to seed size and rodent body size, supporting our hypothesis. Larger *N. confucianus* preferred hoarding and consuming large seeds of *Q. variabilis* and *L. harlandii*, whereas smaller *A. draco* preferred smaller seeds of *C. oleifera* . These findings indicate that functional traits—specifically size traits of rodents and seeds—are key determinants of interaction outcomes in seed–rodent systems.

Functional trait diversity is increasingly recognized as a critical factor shaping mutualism or predation structures in ecosystems (Albrecht et al. 2018). In seed–rodent systems, the balance between mutualism and predation is determined by rodent behavior like scatter‐hoarding, larder‐hoarding, or consumption (Vander Wall 1990; Schupp et al. 2010; Yang et al. 2020). These behaviors are influenced by functional traits of both seeds and rodents, including seed size (Stapanian and Smith 1978; Jansen et al. 2002; Vander Wall 2003; Wang and Chen 2009; Cui et al. 2023), seed coat thickness (Jacobs 1992; Zhang and Zhang 2008; Yang et al. 2023), nutrition (Smallwood and Peters 1986; Chang and Zhang 2014), defensive compounds (Xiao et al. 2022; Nelson et al. 2023), and rodent body size (Zhang et al. 2015; Cheng et al. 2024). For instance, variation in seed size has been shown to drive differential predation by mammals in neotropical rainforests (Dirzo et al. 2007). Several of the previous studies exploring how functional traits influence the balance between mutualism and predation in seed–rodent interactions have been conducted in artificial settings, such as enclosures (Chang and Zhang 2014; Zhang et al. 2016; Niu et al. 2020). For instance, Zhang et al. (2015) found that large rodents interact with both large‐sized and small‐sized seeds, whereas small rodents only interact with small‐sized seeds, though their results showed less distinct preference differentiation compared with ours. Beyond factors like enclosure space limitations and spatially intensified interspecific competition (e.g., increased seed pilferage due to restricted environments), one possible explanation lies in differing experimental contexts (Wang, Wang, and Chen 2024; Yang, Zhao, and Zhang 2025). Their study was carried out in the deciduous broad‐leaved forest of North China, which produced many fewer seed species with seed abundance in a more marked seasonal dynamic. The limited seed resources and harsh winter forced large‐sized rodents to broaden their preference for seed size. Nonetheless, their work, like ours, highlights the role of size traits in structuring seed–rodent interactions.

This study reveals key ecological patterns that advance understanding of trait‐mediated seed–rodent mutualism: the smaller *A. draco* exhibited more frequent scatter‐hoarding behavior, a mutualistic act that disproportionately benefited small‐seeded *C. oleifera* , enhancing its chances of seedling establishment compared with larger‐seeded species. In contrast, the larger *N. confucianus* invested more in scatter‐hoarding large‐seeded *Q. variabilis* and *L. harlandii*, highlighting how rodent body size aligns with seed size to shape which plant species gain dispersal benefits. These patterns underscore that size traits act as “matchmakers” for seed–rodent mutualism, a finding with implications for predicting how plant‐rodent interactions might shift under environmental change (e.g., rodent community turnover). This suggests that handling time—strongly influenced by rodent and seed size—shapes rodent preferences and hoarding benefits, supporting the general argument that trait diversity of consumers and resources determines mutualistic or predatory outcomes in ecological networks (Hillebrand and Matthiessen 2009; Vazquez et al. 2009; Albrecht et al. 2018).

Notably, size is not the sole trait influencing seed selection. For instance, *C. oleifera* seeds are small but also low in tannins (Table 1), whereas *L. harlandii* seeds are large with moderate tannins. Larger rodents may be better equipped to handle tannins, which could partially explain *N. confucianus* 's ability to exploit *L. harlandii* seeds. However, our data showed that size is the primary driver: *A. draco* avoided the largest seeds (*L. harlandii*) despite their moderate tannins, whereas *N. confucianus* preferred large seeds regardless of tannin content. This suggests that size constraints override other traits in shaping these interactions (Wang and Chen 2009), although future studies should explore multi‐trait combinations. A key unresolved question from our hypothesis—why *L. harlandii* (the largest seeds with the thickest coat) was not the main species consumed by *N. confucianus* —can be explained by integrating two trait‐related trade‐offs. Firstly, the extremely thick seed coat of *L. harlandii* (1.43 ± 0.20 mm) significantly extends handling time— *N. confucianus* requires more energy to crack open the coat compared with *Q. variabilis* (0.56 ± 0.09 mm coat thickness), undermining the energetic benefit of its larger size (Jacobs 1992). Alternatively, *L. harlandii* has the lowest fat content (0.58%) among the four‐seed species, and its starch content (53.7%) is only slightly higher than *Q. variabilis* (49.50%), two aspects that indicate comparatively low palatability (Table 1). For *N. confucianus* (53.1 ± 11.5 g), which needs higher energy intake to maintain its larger body mass, *Q. variabilis* offers a more balanced trade‐off: moderate size, thinner coat, lower tannins (6.69% vs. 0.97% of *L. harlandii*), and comparable starch, resulting in higher foraging efficiency per unit time. This aligns with optimal foraging theory, where rodents prioritize energy return relative to handling costs rather than single traits like size alone (Smallwood and Peters 1986; Chang and Zhang 2014).

Sympatric seed predators and dispersers with similar food niches often compete intensely (Zhang et al. 2016). The size‐related differentiation preference we observed likely reduces interspecific competition between *N. confucianus* and *A. draco* , thereby facilitating the coexistence of sympatric rodent species in their natural ecosystem. The main factor driving their differentiation could be the handling time of seeds by rodents. The large size, high weight, and tough husks of *L. harlandii* acorns increase seed‐handling time for small rodents, which would then increase their predation risk from owls, masked palm civets ( *Paguma larvata* ), and even some larger rats (e.g., *Leopoldamys edwardsi* ). Previous studies also show that *L. harlandii* acorns are primarily consumed and dispersed by large rodents (Xiao and Zhang 2006; Wang et al. 2023). Although *L. hancei* seeds are smaller than *L. harlandii*'s, their tough husks may deter both rodent species, reducing harvest rates. The lack of significant pilferage benefit differences between rodents for most seed species suggests that initial harvest and hoarding preferences reduce interspecific pilferage, further easing competition.

Intra‐specific rodent size variation was not a focus of this study, but future work could explore whether individual size differences within species influence seed preferences. Assuming minimal body size/weight changes during our study, such variation might drive additional niche partitioning, but our data indicate that interspecific size differences are the primary structuring force.

The double‐duplex PIT tagging method, previously used to study interactions between two‐seed and two‐rodent species (Gu et al. 2021), was successfully extended here to a four‐seed, two‐rodent system. This demonstrates the method's utility for disentangling complex multi‐partner ecological networks, offering high accuracy in tracking individual‐level interactions—a major advance over traditional methods with limited resolution.

One caveat of our study is the lack of data on seed dispersal outcomes by untagged rodents, which prevents direct comparison with tagged individuals. However, we minimized potential tagging biases by using ultra‐light PIT tags (< 0.1 g) that represent < 0.6% of the body mass of our smallest target rodent ( *A. draco* ). This weight ratio is well below the threshold (1%–2% of body mass) shown to affect small mammal behavior (Korslund and Steen 2006), and post‐tagging monitoring confirmed that there were no abnormal foraging or dispersal behaviors in tagged individuals. Future studies could incorporate a mixed design (tracking both tagged and untagged rodents via camera traps + PIT detection) to explicitly test for tagging effects, though our current measures suggest such effects are negligible for our dataset.

## 5 Conclusion

Our study used individual‐based tracking with double‐duplex PIT tagging to reveal that functional traits—specifically seed size and rodent body size—are primary drivers of seed fates and interaction patterns in the seed–rodent system of a subtropical forest. Larger rodents prefer large seeds, whereas smaller rodents focus on small seeds, with size‐related behaviors reducing interspecific competition and structuring mutualistic‐predatory balances. These findings have two key implications: first, they reinforce that functional trait diversity—specifically matching between rodent body size and seed size—is a cornerstone of maintaining balanced seed–rodent interactions, which in turn sustain forest regeneration by supporting the dispersal of multiple tree species. Second, they validate the double‐duplex PIT tagging technique as a powerful tool for unraveling individual‐level interactions in complex multispecies systems—a method that can be applied to other plant–animal mutualisms (e.g., frugivory) to address longstanding ecological questions. Together, our results advance both theoretical understanding of trait‐mediated interactions and methodological capacity for studying complex ecological networks.

## Author Contributions

**Haifeng Gu:** conceptualization (equal), data curation (equal), investigation (equal), methodology (equal), writing – original draft (equal), writing – review and editing (equal). **Xifu Yang:** investigation (equal), methodology (equal), project administration (equal), writing – original draft (equal), writing – review and editing (equal). **Rodolfo Dirzo:** methodology (equal), writing – review and editing (equal). **Zhibin Zhang:** conceptualization (lead), project administration (equal), supervision (equal), writing – original draft (equal), writing – review and editing (equal).

## Conflicts of Interest

The authors declare no conflicts of interest.

## Supporting information


**Appendix S1:** ece372409‐sup‐0001‐AppendixS1.docx.

## Data Availability

All raw data and the R code used for statistical analyses are available from the Figshare Repository: https://doi.org/10.6084/m9.figshare.29815622.v2 (Gu et al. 2025).
