# Supplementary material for: Functional Traits Shape Seed–Rodent Interactions in a Subtropical Forest: Insights From Individual‐Based Tracking With Double‐Duplex PIT Tagging
Source: Ecol Evol. 2025 Oct 30;15(11):e72409. doi: 10.1002/ece3.72409 (PMC12575268; doi:10.1002/ece3.72409)
Supplement: Supplementary file 1 — Appendix S1: ece372409‐sup‐0001‐AppendixS1.docx. [file ECE3-15-e72409-s001.docx]

**Supplementary materials**

**Functional traits shape seed-rodent interactions in a subtropical forest: Insights from individual-based tracking with double-duplex PIT tagging**

**Table S1** Body mass and trapping success of the three rodent species in the study.

| Rodent species | Scientific name | Number of tagged individuals | Body mass  (mean ± SD, g) | Trapping success rate (%) |
| --- | --- | --- | --- | --- |
| South China field mouse | *Apodemus draco* | 13 | 18.1 ± 4.6 | 3.93 |
| Chinese white-bellied rat | *Niviventer confucianus* | 19 | 53.1 ± 11.5 | 6.40 |
| Chestnut white-bellied rat | *Niviventer fulvescens* | 2 | 56.9 ± 7.5 | 0.44 |

**Table S2** Pearson's Chi-square tests for the preference of seed harvesting (IHS), scatter-hoarding (SH_I_ and SH_F_), and consumption (E) between two sympatric rodent species: *Apodemus draco* (Ad) and *Niviventer confucianus* (Nc). Co = *Camellia oleifera*, Qv = *Quercus variabilis*, Lhar = *Lithocarpus harlandii*, Lhan = *L. hancei*. The numbers in front of the parentheses indicate the observed values, and those in the parentheses indicate theoretical values.

|  |  | Co | Qv | Lhar | Lhan | χ^2^ | *P* |
| --- | --- | --- | --- | --- | --- | --- | --- |
| IHS | Ad | 100 (50.25) | 45 (50.25) | 0 (50.25) | 56 (50.25) | 100.71 | < 0.001 |
|  | Nc | 53 (61.25) | 87 (61.25) | 75 (61.25) | 30 (61.25) | 30.97 | < 0.001 |
| SH_I_ | Ad | 27 (12) | 11 (12) | 0 (12) | 10 (12) | 31.17 | < 0.001 |
|  | Nc | 5 (21.25) | 54 (21.25) | 15 (21.25) | 11 (21.25) | 69.68 | < 0.001 |
| SH_F_ | Ad | 30 (12.5) | 9 (12.5) | 0 (12.5) | 11 (12.5) | 38.16 | < 0.001 |
|  | Nc | 2 (20.75) | 56 (20.75) | 15 (20.75) | 10 (20.75) | 83.99 | < 0.001 |
| E | Ad | 59 (29.75) | 29 (29.75) | 0 (29.75) | 31 (29.75) | 58.58 | < 0.001 |
|  | Nc | 42 (28.5) | 22 (28.5) | 40 (28.5) | 10 (28.5) | 24.53 | < 0.001 |

**Table S3** Estimation of pilferage rate (CPB, IPB, TPB) exerted by two sympatric rodent species: *Apodemus draco* (Ad) and *Niviventer confucianus* (Nc) on harvesting four seed species: *Camellia oleifera* (Co), *Quercus variabilis* (Qv), *Lithocarpus harlandii* (Lhar) and *L. hancei* (Lhan). The numbers in front of parentheses indicate proportions of related parameters. CPB = the benefits from conspecific pilferage. IPB = the benefits from interspecific pilferage. TPB = the benefits from all the pilferage. *n*, sample size. N/A, not applicable.

| Seeds | Rodents | CPB | IPB | TPB |
| --- | --- | --- | --- | --- |
| Co | Ad | 13.3 (*n* = 30) | 16.7 (*n* = 30) | 30.0 (*n* = 30) |
|  | Nc | 0 (*n* = 2) | 100 (*n* = 2) | 100 (*n* = 2) |
| Qv | Ad | 55.6 (*n* = 9) | 11.1 (*n* = 9) | 66.7 (*n* = 9) |
|  | Nc | 23.2 (*n* = 56) | 5.4 (*n* = 56) | 28.6 (*n* = 56) |
| Lhar | Ad | N/A (*n* = 0) | N/A (*n* = 0) | N/A (*n* = 0) |
|  | Nc | 33.3 (*n* = 15) | 0 (*n* = 15) | 33.3 (*n* = 15) |
| Lhan | Ad | 36.4 (*n* = 11) | 9.1 (*n* = 11) | 45.5 (*n* = 11) |
|  | Nc | 40.0 (*n* = 10) | 0 (*n* = 10) | 40.0 (*n* = 10) |

**Table S4** The 2×2 crosstabs with Pearson's Chi-square tests or Fisher’s exact tests for the comparisons of scatter-hoarding benefits (SHB_1_ and SHB_2_) and pilferage benefits (CPB, IPB, TPB) between two sympatric rodent species: *Apodemus draco* (Ad) and *Niviventer confucianus* (Nc). Co = *Camellia oleifera*, Qv = *Quercus variabilis*, Lhan = *L. hancei*. CP = conspecific pilfered seeds. IP = interspecific pilfered seeds. TP = total pilfered seeds. non-CP = SH_F_ – CP. non-IP = SH_F_ – IP. non-TP = SH_F_ – TP. The numbers in front of the parentheses indicate the observed values, and those in the parentheses indicate theoretical values.

|  |  |  | Ad | Nc | *∑* | χ^2^ | *P* |
| --- | --- | --- | --- | --- | --- | --- | --- |
| Co | CPB | CP | 4 (3.75) | 0 (0.25) | 4 |  | 1.000 |
|  |  | non-CP | 26 (26.25) | 2 (1.75) | 28 |  |  |
|  |  | *∑* | 30 | 2 | 32 |  |  |
|  | IPB | IP | 5 (6.6) | 2 (0.4) | 7 |  | 0.042 |
|  |  | non-IP | 25 (23.4) | 0 (1.6) | 25 |  |  |
|  |  | *∑* | 30 | 2 | 32 |  |  |
|  | TPB | TP | 9 (10.3) | 2 (0.7) | 11 |  | 0.111 |
|  |  | non-TP | 21 (19.7) | 0 (1.3) | 21 |  |  |
|  |  | *∑* | 30 | 2 | 32 |  |  |
| Qv | CPB | CP | 5 (2.5) | 13 (15.5) | 18 |  | 0.101 |
|  |  | non-CP | 4 (6.5) | 43 (40.5) | 47 |  |  |
|  |  | *∑* | 9 | 56 | 65 |  |  |
|  | IPB | IP | 1 (0.6) | 3 (3.4) | 4 |  | 0.458 |
|  |  | non-IP | 8 (8.4) | 53 (52.6) | 61 |  |  |
|  |  | *∑* | 9 | 56 | 65 |  |  |
|  | TPB | TP | 6 (10.3) | 16 (0.7) | 22 | 3.47 | 0.063 |
|  |  | non-TP | 3 (19.7) | 40 (1.3) | 43 |  |  |
|  |  | *∑* | 9 | 56 | 65 |  |  |
| Lhan | CPB | CP | 4 (4.2) | 4 (3.8) | 8 |  | 1.000 |
|  |  | non-CP | 7 (6.8) | 6 (6.2) | 13 |  |  |
|  |  | *∑* | 11 | 10 | 21 |  |  |
|  | IPB | IP | 1 (0.5) | 0 (0.5) | 1 |  | 1.000 |
|  |  | non-IP | 10 (10.5) | 10 (9.5) | 20 |  |  |
|  |  | *∑* | 11 | 10 | 21 |  |  |
|  | TPB | TP | 5 (4.7) | 4 (4.3) | 9 |  | 1.000 |
|  |  | non-TP | 6 (6.3) | 6 (5.7) | 12 |  |  |
|  |  | *∑* | 11 | 10 | 21 |  |  |
